# Clinical characteristics of intracranial aneurysms in elderly patents over 70 years old: a retrospective observational study

**DOI:** 10.1186/s12883-022-02786-z

**Published:** 2022-07-11

**Authors:** Ruiqi Chen, Dingke Wen, Anqi Xiao, Rui Guo, Chao You, Yi Liu

**Affiliations:** Department of Neurosurgery, West China Hospital, Sichuan University, No. 37 Guo Xue Xiang, Chengdu, Sichuan 610041 P.R. China

**Keywords:** Intracranial aneurysms, Elderly patients, Clinical characteristics, Craniotomy treatment, Endovascular treatment

## Abstract

**Background:**

Although the characteristics of intracranial aneurysms (IAs) in different age groups have been well documented, they remain relatively unclear in elderly patients due to a lack of large sample studies.

**Methods:**

Data from IA patients aged more than 70 years who were treated in our centre from January 2016 to January 2020 were retrospectively collected.

**Results:**

A total of 290 elderly patients (75.9% female) with a mean age of 74.0 ± 4.7 years were analysed. Rupture occurred in 60.7% of patients, 38.6% of whom presented with meningeal irritation, and seizures were noted in 2.3%. A total of 48.9% of the patients with ruptured IAs had initial symptoms presenting with slow development, and the mean delay from ictus was prolonged to 264.2 ± 914.0 hours. In addition, 61.9% of the patients with ruptured IAs had lesions with a maximum diameter of less than 5 mm. A total of 30.3% of the patients had multiple aneurysms, 35.5% had aneurysms with irregular shapes and 54.8% had cerebrovascular atherosclerotic stenosis (CAS). Pulmonary infection (*n* = 138, 47.6%), hydrocephalus (*n* = 72, 24.8%), and thrombosis (*n* = 35, 12.1%) were common complications during hospitalization. By the end of the 1-year follow-up, 22.1% of the patients had unfavourable clinical outcomes, and the mortality rate was 23.4%.

**Conclusions:**

Several characteristics regarding IAs in elderly patients were reported, including an obvious female predominance; mild, slow initial symptom development causing prolonged admission delay; a low incidence of meningeal irritation and seizures due to decreased electrophysiological activity of the neurons; increased percentages of CAS, multiple aneurysms, and aneurysms with daughter sacs causing a high risk of rupture even for small lesions; a high risk of complications during hospitalization; and relatively poor clinical outcomes.

## Background

Intracranial aneurysm (IA) is a devastating cerebral vascular disease and a major threat to health worldwide. According to previous epidemiological reports, unruptured IAs are found in approximately 3.2% of the adult population worldwide, with the average annual incidence of IA-related subarachnoid haemorrhage (SAH) among people of all ages ranging from 6 to 27.63 per 100,000 persons [1, 2]. With the general ageing of the population and advances in neuroimaging diagnosis, an increasing number of elderly patients are being diagnosed with IA [3, 4]. A Japanese cohort study on the natural course of cerebral aneurysms reported that 28% of patients with unruptured IAs were older than 70 years [5]. However, due to a lack of large-sample studies, the overall clinical characteristics of IAs in elderly patients have yet to be fully elucidated. The present study was conducted to clarify the clinical characteristics of elderly patients (more than 70 years old) with IAs, to reveal some new findings and to assist in the choice of best clinical decision for these patients.

## Methods

### Study design

We performed a retrospective analysis in a single tertiary centre. This study was approved by the institutional review board (IRB) of West China Hospital. We used the hospital information system to collect the related medical data of elderly patients with IAs from January 2016 to January 2020. The inclusion criteria were as follows: 1) IA as identified by discharge diagnoses using the key words “intracranial aneurysm” or “aneurysmal subarachnoid haemorrhage”, IAs were diagnosed through computed tomography angiography (CTA) or digital subtraction angiography (DSA), and SAH was confirmed by the results of initial computerized tomography (CT) scan by experienced neuroimaging specialists; and 2) age more than 70 years. Two experienced neurosurgeons and two neuroradiologists who were blinded to the medical records were invited to confirm the diagnoses of the selected patients. Patients with an incorrect diagnosis or incomplete medical profiles were excluded.

### Data collection and grouping

The following patient baseline information was retrieved from the hospital information system: age, sex, smoking, alcohol consumption, history of hypertension (treatment), anaemia, hypoproteinaemia, diabetes mellitus, electrolyte disturbance, coronary disease, hyperlipidaemia, haemorrhagic or ischaemic stroke, encephalatrophy and pulmonary infection, clinical presentations (including symptoms and signs), Glasgow Coma Scale (GCS) score on admission and upon discharge, Hunt-Hess grade, and duration from ictus to admission. Neuroradiological data were collected from the hospital imaging system to evaluate the angiographic metrics of the IAs, including their size (aneurysms with diameters larger than 25 mm were considered giant, and those between 15 and 25 mm were considered large), morphology and location and the presence of multiple aneurysms, based on the results of CTA or DSA. Vascular anomalies were also recorded, mainly including cerebrovascular atherosclerotic stenosis (CAS) and cerebral artery variants in the posterior cerebral artery (PCA), defined as foetal-type posterior cerebral artery (FTP). Irregular aneurysms were defined as the presence of a daughter sac in addition to the main saccular aneurysm. Treatment approaches were documented, and the occurrence of complications was tracked from the daily medical course record during hospitalization. Follow-up information was documented from clinic visits or telephone interviews at discharge and 1 month, 3 months, 6 months and 1 year after discharge. Patient clinical outcomes were measured by GCS upon discharge and modified Rankin scale (mRS) scores during follow-up. GCS scores ≥13 and mRS scores of 0–2 were considered favourable outcomes. The patients were divided into ruptured and unruptured groups, and comparisons were made between the two groups.

### Statistical analysis

SPSS statistical software (version 22.0; SPSS Inc., Chicago, Illinois, USA) was used for all statistical analyses. The mean ± standard deviation (SD) was reported for quantitative data. Categorical data are expressed as frequencies and percentages. Bivariate analyses were conducted using the chi-square test or Fisher’s exact tests, Student’s t-tests, and Mann–Whitney U-tests as appropriate. Significance was defined as *P* < 0.05, and 95% confidence intervals (CIs) were calculated for each variable.

## Results

### Baseline characteristics

A total of 2730 patients with IAs were treated in our hospital from January 2016 to January 2020; among them, 290 elderly patients (≥70 years old on admission) (10.6%) were analysed in the current study. The mean age of the patients was 74.0 ± 4.7 years (range 70 to 95 years old); a total of 220 (75.9%) patients were female. Patient demographic information and history of previous illness are shown in Table 1. Compared with patients in the unruptured group (*n* = 114, 39.3%), patients in the ruptured group (*n* = 176, 60.7%) were significantly older (*P* < 0.001) and had a significantly higher percentage of patients with hypoproteinaemia (*P* < 0.001), anaemia (*P* < 0.001), diabetes mellitus (*P* < 0.001), electrolyte disturbance (*P* = 0.001), pulmonary infection (*P* = 0.001), coronary disease (*P* = 0.005), brain atrophy (*P* = 0.032) and ischaemic stroke (*P* = 0.009). The unruptured group had a significantly higher percentage of patients receiving routine treatment for hypertension than the ruptured group (*P* = 0.024).

**Table 1:** Baseline information of elderly patients with intracranial aneurysms

Characteristics	All combined (*n* = 290)	Ruptured (*n* = 176)	Unruptured (*n* = 114)	*P* value
Age (SD)/years old	74.0 ± 4.7	74.5 ± 4.8	73.3 ± 4.3	0.023
Female patients, (%)	220/290 (75.9)	132/176 (75.0)	88/114 (77.2)	0.670
Smoking, (%)	25/290 (8.6)	15/176 (8.5)	10/114 (8.8)	0.941
Alcohol consumption, (%)	15/290 (5.2)	6/176 (3.4)	9/114 (7.9)	0.158
Hypertension, (%)	228/290 (78.6)	144/176 (81.8)	84/114 (73.7)	0.133
Regular treatment of hypertension, (%)	198/228 (86.8)	119/144 (82.6)	79/84 (94.0)	0.024
Hypoproteinaemia, (%)	171/290 (59.0)	130/176 (73.9)	41/114 (36.0)	< 0.001
Anaemia, (%)	152/290 (52.4)	119/176 (67.6)	33/114 (28.8)	< 0.001
Diabetes mellitus, (%)	156/290 (53.8)	133/176 (75.6)	23/114 (20.2)	< 0.001
Electrolyte disturbance, (%)	143/290 (49.3)	113/176 (64.2)	30/114 (26.3)	< 0.001
Hyperlipidaemia, (%)	36/290 (12.4)	17/176 (9.7)	19/114 (16.7)	0.113
Pulmonary infection, (%)	110/290 (37.9)	80/176 (45.5)	30/114 (26.3)	0.001
Coronary disease, (%)	38/290 (13.1)	31/176 (17.6)	7/114 (6.1)	0.005
Brain atrophy, (%)	32/290 (11.0)	25/176 (14.2)	7/114 (6.1)	0.032
Ischaemic stroke, (%)	98/290 (33.8)	72/176 (40.9)	26/114 (22.8)	0.009
Haemorrhagic stroke, (%)	8/290 (2.8)	6/176 (3.4)	2/114 (1.8)	0.636

Elderly patients: age ≥ 70 years old; *SD* standard deviation

### Clinical presentation

#### Elderly patients with ruptured aneurysms

For the 176 (60.7%) patients who had ruptured aneurysms, the attacks commonly occurred in the morning after waking (*n* = 49, 27.8%). Common inducements included straining during defecation (*n* = 24, 13.6%), emotional fluctuation (*n* = 15, 8.5%) and sneezing (*n* = 8, 4.5%). Presentations included headache (*n* = 148, 84.1%), vomiting (*n* = 118, 67.0%), loss of consciousness (*n* = 64, 36.4%), dizziness (*n* = 22, 12.5%) and hemiparesis (*n* = 20, 11.4%), while only 4 patients (2.3%) presented with seizures. Meningeal irritation (*n* = 68, 38.6%) was the most common sign, followed by cranial nerve deficits (*n* = 22, 12.5%). Slow, progressive development of initial symptoms was documented in 86 (48.9%) patients rather than the typical pattern of sudden onset. Consequently, the mean delay from ictus to admission was prolonged to 264.2 ± 914.0 hours. On admission, a total of 130 patients (73.9%) had good clinical performance (Hunt-Hess grade I-III) (Table 2).

**Table 2:** Clinical presentations of elderly patients with ruptured intracranial aneurysms

Characteristics	All combined	%
Ruptured patients	176/290	60.7
Symptoms slow development	86/176	48,9
Headache	148/176	84.1
Unconsciousness	64/176	36.4
Vomiting	118/176	67.0
Dizziness	22/176	12.5
Hemiparesis	20/176	11.4
Seizure	4/176	2.3
Meningeal irritation	68/176	38.6
Cranial nerve deficit	22/176	12.5
Admission delay (SD)/hours	264.2 ± 914.0	–
GCS (SD)	11.9 ± 4.1	–
Good clinical presentation	130/176	73.9

Elderly patients: age ≥ 70 years old; *SD* standard deviation; *GCS* Glasgow Coma Score; Good clinical presentation: Hunt-Hess grade (I-III)

#### Elderly patients with unruptured aneurysms

Among the remaining 114 (39.3%) patients with unruptured aneurysms, dizziness was the most common symptom (*n* = 42, 36.8%), followed by headache (*n* = 24, 21.1%), limb weakness (*n* = 14, 12.3%) and vomiting (*n* = 6, 5.3%). The most common sign was cranial nerve deficits (*n* = 28, 24.6%) (Table 3).

**Table 3:** Clinical presentations of elderly patients with unruptured intracranial aneurysms

Characteristics	All combined	%
Unruptured patients	114/290	39.3
Dizziness	42/114	36.8
Headache	24/114	21.1
Limb weakness	14/114	12.3
Vomiting	6/114	5.3
Cranial nerve deficit	28/114	24.6

Elderly patients: age ≥ 70 years old; *SD* standard deviation

### Radiological characteristics

Other radiological characteristics of the patients are shown in Table 4. The distribution of modified Fisher grade for the 176 ruptured cases (60.7%) showed that grades 3 and 4 accounted for 60.8% (*n* = 107) of all ruptured cases. Multiple aneurysms were diagnosed in 88 patients (30.3%). The mean size of the aneurysms (largest diameter) was 5.8 ± 4.6 mm. Notably, of the 176 patients with ruptured lesions, 109 (61.9%) had lesions with a largest diameter of less than 5 mm. In addition, 103 (35.5%) patients had irregular lesions with daughter sacs. Furthermore, CAS, brain atrophy and FTP were recorded in 159 (54.8%), 32 (11.0%) and 39 (13.4%) patients, respectively.

**Table 4:** Radiological characteristics of elderly patients with intracranial aneurysms

Characteristics	All combined (*n* = 290)	Ruptured (*n* = 176)	Unruptured (*n* = 114)	*P* value
SAH + ICH, (%)	–	29/176 (16.5)	–	–
Modified Fisher grade I-II, (%)	–	69/176 (39.2)	–	–
Modified Fisher grade III-IV, (%)	–	107/176 (60.8)	–	–
With irregular aneurysms, (%)	103/290 (35.5)	88/176 (50.0)	15/114 (13.2)	< 0.001
CAS, (%)	159/290 (54.8)	121/176 (68.8)	38/114 (33.3)	< 0.001
FTP, (%)	39/290 (13.4)	33/176 (18.8)	6/114 (5.3)	0.001
With multiple aneurysms, (%)	88/290 (30.3)	51/176 (29.0)	37/114 (32.5)	0.529
Dissecting aneurysms, (%)	12/290 (4.1)	7/176 (4.0)	5/114 (4.4)	0.896
Lesion diameter (SD)/mm	5.8 ± 4.6	5.1 ± 3.3	6.8 ± 6.0	< 0.001
Small-size aneurysm, (%)	158/290 (54.5)	113/176 (64.2)	45/114 (39.5)	< 0.001
Anterior circulation, (%)	280/290 (96.6)	172/176 (97.7)	108/114 (94.7)	0.301
ICA, (%)	204/290 (70.3)	124/176 (70.5)	80/114 (70.2)	0.959
MCA, (%)	58/290 (20.0)	38/176 (21.6)	20/114 (17.5)	0.400
ACA, (%)	24/290 (8.3)	18/176 (10.2)	6/114 (5.3)	0.134
Acom, (%)	32/290 (11.0)	18/176 (10.2)	14/114 (12.3)	0.586
Posterior circulation, (%)	24/290 (8.3)	10/176 (5.7)	14/114 (12.3)	0.076
PCA, (%)	4/290 (1.4)	2/176 (1.1)	2/114 (1.8)	0.940
PICA, (%)	2/290 (0.7)	2/176 (1.1)	0/114 (0)	0.678
VA, (%)	14/290 (4.8)	5/176 (2.8)	9/114 (7.9)	0.093
BA, (%)	4/290 (1.4)	1/176 (0.6)	3/114 (2.6)	0.339

Elderly patients: age ≥ 70 years old; *SAH* subarachnoid haemorrhage; *ICH* intracerebral haemorrhage; *CAS* cerebrovascular atherosclerotic stenosis; *FTP* foetal-type posterior cerebral artery; *SD* standard deviation; *ICA* internal carotid artery; *MCA* middle cerebral artery; *ACA* anterior cerebral artery; *Acom* anterior communicating artery; *PCA* posterior cerebral artery; *PICA* posterior inferior cerebellar artery; *VA* vertebral artery; *BA* basilar artery

Compared with the unruptured group, the ruptured group had significantly higher percentages of patients with irregular aneurysms (*P* < 0.001), CAS (*P* < 0.001), and FTP (*P* = 0.001). In addition, the mean lesion diameter in the ruptured group was significantly smaller (*P* < 0.001), and the percentage of patients with small aneurysms was significantly higher than that in the unruptured group (*P* < 0.001).

### Treatment and complications during hospitalization

The patients’ treatment modalities and incidence of complications are shown in Table 5. A total of 181 patients (62.4%) were treated by surgical intervention, including 91 (31.4%) with craniotomy clipping and 90 (31.0%) with an endovascular approach; the remaining 109 patients (37.6%) received conservative treatment. Pulmonary infection (*n* = 138, 47.6%), hydrocephalus (*n* = 72, 24.8%), and thrombosis (*n* = 35, 12.1%) were common complications during hospitalization. Notably, some commonly seen complications in other age groups, such as seizures (1.7%) and vasospasm (2.8%), were not obvious in our series.

**Table 5:** Treatment, complications and prognosis of elderly patients with intracranial aneurysms

Characteristics	All combined (*n* = 290)	Ruptured (*n* = 176)	Unruptured (*n* = 114)	*P* value
Craniotomy, (%)	91/290 (31.4)	75/176 (42.6)	16/114 (14.0)	< 0.001
Endovascular, (%)	90/290 (31.0)	33/176 (18.8)	57/114 (50.0)	< 0.001
Conservative, (%)	109/290 (37.6)	68/176 (38.6)	41/114 (36.0)	0.646
Pulmonary infection, (%)	138/290 (47.6)	83/176 (47.2)	55/114 (48.2)	0.856
Hydrocephalus, (%)	72/290 (24.8)	43/176 (24.4)	29/114 (25.4)	0.846
Thrombosis, (%)	24/290 (12.1)	20/176 (11.4)	4/114 (3.5)	0.031
Rebleeding, (%)	23/290 (7.9)	14/176 (8.0)	9/114 (7.9)	0.985
Gastrointestinal bleeding, (%)	16/290 (5.5)	8/176 (4.5)	8/114 (7.0)	0.368
Seizure, (%)	5/290 (1.7)	2/176 (1.1)	3/114 (2.6)	0.622
Blood vasospasm, (%)	8/290 (2.8)	4/176 (2.3)	4/114 (3.5)	0.794
Intracranial infection, (%)	15/290 (5.2)	9/176 (5.1)	6/114 (5.3)	0.955

Elderly patients: age ≥ 70 years old

For the comparison between the two groups, the ruptured group had significantly higher percentages of patients who underwent craniotomy surgery (*P* < 0.001), while the unruptured group had a significantly higher ratio of cases undergoing the endovascular approach (*P* < 0.001). Regarding complications, the ruptured group had a significantly higher percentage of patients with thrombosis than the unruptured group (*P* = 0.031).

### Clinical outcomes

Patient clinical outcomes are shown in Table 6. The mean length of hospital stay was 9.5 ± 10.7 days, ranging from 1 to 74 days. By the time of discharge, favourable clinical outcomes (GCS score ≥ 13) were seen in 199 (68.6%) patients. A total of 35 patients (12.1%) died during their stay in the hospital. By the end of the 1-year follow-up, 158 patients (54.5%) demonstrated favourable outcomes (mRS score 0–2), while 33 additional patients had died, resulting in a total mortality rate of 23.4% (*n* = 68). The remaining 64 (22.1%) patients demonstrated unfavourable outcomes (mRS score 3–5)**.**

**Table 6:** Clinical outcomes of elderly patients with intracranial aneurysms

Characteristics		All combined (*n* = 290)	Ruptured (*n* = 176)	Unruptured (*n* = 114)	*P* value
Mean length of stay (SD)/day		9.5 ± 10.7	10.5 ± 11.1	7.8 ± 9.9	0.037
Discharge	Favourable, (%)	199/290 (68.6)	97/176 (55.1)	102/114 (89.5)	< 0.001
	Unfavourable, (%)	56/290 (19.3)	49/176 (27.8)	7/114 (6.1)	< 0.001
	Death, (%)	35/290 (12.1)	30/176 (17.0)	5/114 (4.4)	0.002
3 month	Favourable, (%)	187/290 (64.5)	87/176 (49.4)	100/114 (87.7)	< 0.001
	Unfavourable, (%)	61/290 (21.0)	53/176 (30.1)	8/114 (7.0)	< 0.001
	Death, (%)	42/290 (14.5)	36/176 (20.5)	6/114 (5.3)	< 0.001
6 month	Favourable, (%)	164/290 (56.6)	75/176 (42.6)	89/114 (78.1)	< 0.001
	Unfavourable, (%)	71/290 (24.5)	56/176 (31.8)	15/114 (13.2)	< 0.001
	Death, (%)	55/290 (19.0)	45/176 (25.6)	10/114 (8.8)	< 0.001
1-year follow-up	Favourable, (%) outcomes	158/290 (54.5)	72/176 (40.9)	86/114 (75.4)	< 0.001
	Unfavourable, (%)	64/290 (22.1)	46/176 (26.1)	18/114 (15.8)	0.038
	Death, (%)	68/290 (23.4)	58/176 (33.0)	10/114 (8.8)	< 0.001

Elderly patients: age ≥ 70 years old; *SD* standard deviation; *GCS* Glasgow Coma Score; Favourable outcomes: GCS score ≥ 13 at discharge and Modified Rankin Scale of 0–2 during follow-up; Unfavourable outcomes: GCS score < 13 at discharge and Modified Rankin Scale of 3–6 during follow-up

The clinical outcomes showed significant differences between the ruptured and unruptured groups. Patients in the ruptured group had a significantly longer length of hospital stay than those in the unruptured group (*P* = 0.037). Additionally, the ruptured group had significantly better clinical outcomes at different follow-up time points, presenting as significantly higher percentages of unfavourable outcomes (*P* < 0.05) and death (*P* < 0.05) and significantly lower rates of patients who had favourable outcomes (*P* < 0.001).

## Discussion

IAs in the elderly population are not very well documented due to a lack of large-sample studies. In addition, existing studies have mainly focused on patients with unruptured aneurysms [6–9]. Subsequently, the overall characteristics of IAs in elderly patients remain unclear. In the present study, several clinical characteristics of elderly IAs were reported. The findings of our study will be helpful for obtaining a better understanding of IAs in elderly age subgroups and will be beneficial for medical professionals in making the best clinical decision.

### Female predominance

A significant female predominance (75.9%) was noted in our cohort. This result is consistent with some previous studies [8, 10, 11], but ours reported an even higher sex imbalance. Interestingly, this female predominance was not significant according to data from our younger aneurysm patient cohort, and paediatric IA patients even showed a reverse sex predominance [12]. One explanation for this discrepancy is differences in the quantity of oestrogen receptors after menopause, which might result in deterioration of vascular biology and reduced fibrillar collagen in the cerebral arteries [13–15]. This difference is also reported to be responsible for the greater propensity for aneurysm rupture [16]. Admittedly, the generally longer life span in women could also contribute to this observation [17], yet further evidence is needed to validate this assertion.

### Small lesions carry a high risk of rupture

**Fig. 1:**
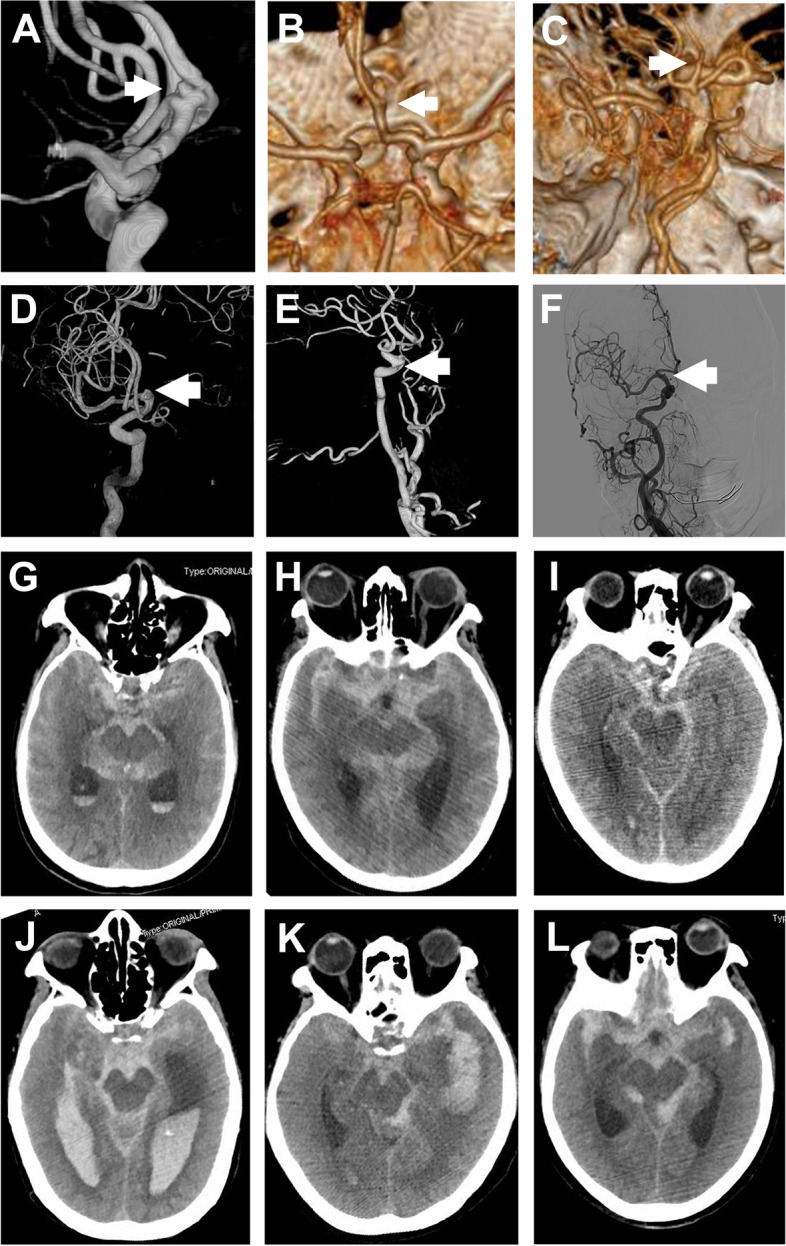
Small ruptured aneurysms with a high modified Fisher grade due to widespread, thick haemorrhage in the expanded subarachnoid space caused by obvious brain atrophy

It has been widely reported that the risk of rupture increases with the size of aneurysms [18]. However, in the present study, it is worth noting that of the 176 patients with ruptured lesions, 109 (61.9%) had lesions with a largest diameter of less than 5 mm, which is smaller than the commonly recognized risk factors for rupture (> 7 mm) [19] and the surgical indication (> 5 mm) [20] based on the study of different age groups. Several factors, particularly in this age group, may contribute to this result. 1) The percentage of patients with a previous disease history, such as hypertension and diabetes mellitus, was higher than that in previous studies on other IA age groups. These comorbidities are reported to be risk factors for the rupture of small IAs [21]. 2) In morphological analysis, we noted a higher percentage of multiple aneurysms and lesions with daughter sacs. These factors have been reported to be associated with a propensity for future rupture [22, 23]. 3) Our present data showed an elevated percentage of CAS and cerebral variants (FTP) in elderly patients, which has been suggested as a possible factor for the development and even rupture of IAs [24, 25]. Together, structural abnormalities related to congenital vascular variants and multiple lesions, vessel injury caused by comorbidities and particular morphological characteristics are very likely to contribute to the formation and rupture of IAs in later stages of life, even for small lesions. Based on our results, more attention should be given to elderly patients with multiple unruptured aneurysms, with daughter sacs and with CAS and FTP, even for lesions measuring less than 0.5 cm. Typical radiological images of ruptured small lesions with diffuse SAH in our elderly patient cohort are shown in Fig. 1.

### Slow development of initial symptoms caused a prolonged admission delay

In our study, we noted that the initial clinical symptoms for elderly patients with IAs were generally milder. Even for patients with ruptured IAs, headache and unconsciousness were more likely to present with slow development. Typical thunderclap headache symptoms observed in other age groups were relatively uncommon [26]; consequently, much of the delay in admission might have resulted from ignorance of early mild symptoms and overlooking the importance of early treatment. A previous study clearly specified that the delay in admission for elderly patients contributed to a poorer prognosis among the patients [27]. We suggest that medical professionals pay more attention to neurological symptoms such as moderate or even mild headache in this cohort. Possible aneurysm rupture should be considered, and a radiological exam should be arranged in a timely manner.

### Complications during hospitalization

#### Decreased electrophysiological activity of neurons causes a low incidence of seizures, blood vasospasm and meningeal irritation

Previous studies based on different age groups noted that seizure onset was commonly associated with rupture of an IA, mainly due to the stimulation of bleeding to the cerebral cortex as well as vasospasm and subsequent ischaemic events [28, 29]. According to our previous study, the incidence of seizures was much higher in paediatric patients who carry an IA located distal to the circle of Willis [30]. For our elderly IA patient cohort, however, the incidence of seizures upon admission was obviously lower than that in previous studies on IAs in different age groups. In addition, we noticed a high modified Fisher grade in our ruptured cases due to widespread, thick haemorrhage in the expanded subarachnoid space due to obvious brain atrophy; in contrast, however, the occurrence of vasospasm was low. Some studies have suggested that arterial ageing significantly influences normal vasoconstriction, and the cellular response towards haemodynamic changes in aged arteries is reduced [31]. Additionally, it is presumed that regional metabolic dysfunction leads to the disrupted release of excitotoxic neurotransmitters [32]. This evidence indicates that ageing and brain atrophy significantly affect the normal regulation of the electrophysiological activity of neurons and arterial component cells, which was also supported by the low incidence of meningeal irritation revealed in our study.

#### High risk of pulmonary infection and thrombosis contribute to poor prognosis

Regardless of the presence of seizures and meningeal irritations, we noticed that the risk of pulmonary infection and thrombosis in our elderly IA patients was greater than that in previous IA studies on different age groups [33, 34]. For elderly patients, slow recovery after treatment, a prolonged length of stay in the hospital and excess time spent lying in bed may contribute to this result. It is worth noting that a certain number of deaths were caused by these complications rather than rupture of the aneurysm itself. Therefore, medical professionals should pay more attention to taking measures to prevent or address these complications during hospitalization.

### Limitations

To the best of our knowledge, the present study is a relatively large single-centre retrospective study that has validated the clinical characteristics and surgical outcomes of elderly patients with IAs to date. The limitations of this study include its nonrandomized, retrospective, observational and single-centre design, as the database only had hospitalized patients, and those with small unruptured lesions or who did not wish to have surgical treatment were not included in the analysis, which may have led to case selection bias. In addition, the characteristics included for analysis were limited due to the scope of the database, and some important factors, such as fluctuation of blood pressure, treatment of hypertension (i.e., type of medication, duration) and correlation with occurrence of rupture, were not included in the present study. Therefore, additional well-designed studies are required to obtain more robust evidence and verify the results presented in this study. In addition, although we performed bivariate analysis to explore differences in the clinical characteristics between the ruptured and unruptured groups, further multiple logistic regression analysis should be conducted to reveal independent factors related to the rupture of IAs in this age group.

## Conclusions

Several findings regarding IAs in elderly patients were reported in this study, including an obvious female predominance; relatively mild initial symptoms causing prolonged admission delay; a low incidence of meningeal irritation, seizures and vasospasm due to decreased electrophysiological activity of neurons; increased percentages of CAS, multiple aneurysms, and aneurysms with daughter sacs causing a high risk of rupture even for small lesions; a high risk of complications during hospitalization; and relatively poor clinical outcomes.

## Data Availability

The datasets used and/or analysed during the current study are available from the corresponding author on reasonable request.

## Abbreviations

IA: Intracranial aneurysm
SAH: Subarachnoid hemorrhage
BA: Basilar artery
GCS: Glasgow Coma Scale
CTA: Computed tomography angiography
SD: Standard deviation
DSA: Digital subtraction angiography
CAS: Cerebrovascular atherosclerotic stenosis
PCA: Posterior cerebral artery
FTP: Fetal-type posterior cerebral artery
mRS: Modified Rankin scale
CI: Confidence interval
CT: Computed tomography
ICH: Intracerebral hemorrhage
ICA: Internal carotid artery
MCA: Middle cerebral artery
ACA: Anterior cerebral artery
Acom: Anterior communicating artery
VA: Vertebral artery
PICA: Posterior inferior cerebellar artery
